# Autobiographical memory specificity and restrained eating: examining the influence of priming with images of healthy and unhealthy foods

**DOI:** 10.1007/s40519-023-01577-w

**Published:** 2023-06-21

**Authors:** Deborah J. Wallis, Jessica Moss, Bethany Varnam, Barbara Dritschel, Nathan Ridout

**Affiliations:** 1 School of Applied Social Sciences, De Montfort University, Leicester, LE1 9BH UK; 2 School of Psychology, University of St Andrews, St Andrews, KY16 9BU UK; 3 School of Psychology, College of Health and Life Sciences, Aston University, Aston Triangle, Birmingham, B4 7ET UK; 4 Present Address: School of Social Sciences, Birmingham City University, Birmingham, B4 7BD UK

**Keywords:** Dieting, Restraint, Memory-specificity, Positive affect, Priming

## Abstract

**Background:**

Dietary restraint has been linked to deficits in the ability to recall detailed memories of personally experienced events (referred to as autobiographical memory specificity). As priming with healthy foods increases the salience of restraint it would be expected to lead to greater deficits in memory specificity.

**Objective:**

To determine if priming word cues with images of healthy or unhealthy foods would influence the specificity of memory retrieval, and if deficits in memory specificity would be more evident in those reporting higher levels of dietary restraint, or currently dieting.

**Methods:**

Sixty female undergraduates self-reported if they were currently dieting and completed measures of mood, restraint, and disinhibition, and a modified version of the autobiographical memory task. Participants were presented with positive and negative words (unrelated to eating concerns) and asked to retrieve a specific memory in response to each cue. A food image was shown prior to each word cue; half of the participants were primed with images of healthy foods and half with images of unhealthy foods.

**Results:**

As expected, participants primed with healthy foods retrieved fewer specific memories than did those primed with unhealthy foods. However, neither restraint nor current dieting behaviour was associated with memory specificity.

**Conclusions:**

Differences in memory specificity between the priming conditions cannot be explained in terms of increased salience of restraint. However, it is plausible that unhealthy images led to an increase in positive affect, which in turn improved memory specificity.

**Level of evidence:**

Level I: Evidence obtained from: at least one properly designed experimental study.

## Introduction

Autobiographical memory specificity (AMS) refers to the recollection of detailed memories of personally experienced events from one’s past and is intimately linked to the experience of the self [1]. Specific memories (e.g., *‘flying for the first time’*) can come to mind spontaneously. However, everyday functioning often requires a voluntary search for memories, which makes demands on cognitive resources, particularly executive function [1]. A great deal of evidence relating to AMS has been generated using the autobiographical memory test (AMT [2];). This task involves presenting participants with a series of cues (normally words) and inviting them to retrieve a specific memory from their past in response to each cue. Specific memories are defined as a memory of an event that occurred at a particular time and place, and that lasted less than one day. Studies using the AMT have revealed that certain groups have difficulties in accessing memories at the specific level. For example, participants with depression retrieve fewer specific memories (< 60% of trials) compared to controls (> 70% of trials) and instead retrieve categorical memories (e.g., “I always used to be happy”) [3]. This finding has been replicated numerous times (See [4] for a review) and has also been observed in individuals who have experienced significant trauma (see [5] for a review). Deficits in memory specificity have also been observed when images were used to cue memories in depressed and traumatised samples [6, 7]. Poor AMS is associated with several negative consequences including poorer prognosis in participants with depression [8], greater risk of suicide [4], and impaired social problem solving [9, 10]. Furthermore, poor AMS can predict depression in response to life stress, even in those without a history of depression [11] and moderate the impact of daily problems on negative mood [12].

There is a growing body of evidence demonstrating impaired AMS in participants with clinical [13–17] and subclinical disordered eating [10, 18]. One plausible explanation for poor AMS in participants with disordered eating relates to dietary restraint, which refers to the intentional restriction of food intake in order to reduce weight. Notably, restraint is associated with deficits in executive function, possibly due to individuals engaging in task irrelevant thoughts concerning food, weight, and body image [19–21]. Given the importance of executive function in voluntary retrieval of autobiographical memories [22, 23], restraint would be expected to impair specificity by reducing the executive resources available to be utilised during the autobiographical memory search. Consistent with this line of reasoning, individuals who were currently dieting have been shown to retrieve fewer specific memories than non-dieters [24]. Similarly, scores on a measure of restraint, particularly *concern with dieting*, have been shown to negatively correlate with memory specificity [25]. There is also evidence that scores on the *drive-for-thinness (DFT)* subscale of the Eating Disorders Inventory (EDI, [26]), which correlates highly with measures of restrained eating [27], negatively predicts memory specificity [10]. Assuming that dietary restraint underlies the deficits in AMS observed in disordered eating, increasing the salience of this factor at the time of memory retrieval should worsen memory specificity.

A robust method of making a concept salient is priming, which refers to the subconscious activation of mental representations (i.e. schema) that then bias the interpretation of incoming information, which in turn influences subsequent behaviour [28]. This is usually achieved by presenting one stimulus (the prime) for a brief period prior to the onset of the target stimulus [29]. In the context of eating, there is evidence that participants ate less after being primed with messages relating to healthy diet compared to a non-primed control group [30]. Similarly, in another study, females consumed fewer calories after being primed with words connoting healthy body image (e.g., slim) than neutral primes (e.g., room) [31]. Interestingly, priming participants with images of healthy snacks (compared to unhealthy) increased restraint in restrained eaters, but not unrestrained eaters [32]. A review of the priming literature confirmed the effectiveness of low-calorie foods in priming restraint [33]. Thus, images of healthy foods would seem the ideal method of priming restraint in the current study, with images of unhealthy foods being used in the control condition.

The aim of the current study was to examine if using healthy and unhealthy food images to prime word cues on an autobiographical memory test would influence the proportion of specific memories retrieved on this task. Participants completed a modified version of the AMT whereby each memory cue (word) was preceded by an image of a food item. Half of the participants were primed with healthy images and half with unhealthy images. Participants reported if they were currently dieting and completed measures of mood (depression and anxiety), disinhibition and restraint. As healthy images were expected to make restraint salient [32, 33] and as restraint has been linked to reduced memory specificity [10, 26], it was predicted that, after controlling for depression, individuals primed with healthy images would retrieve significantly fewer specific memories on the AMT than would participants primed with unhealthy images. However, as priming of restraint should be more evident in those with high restraint scores [32] it was expected that individuals with higher restraint scores in the healthy prime condition would exhibit poorer memory specificity than would those with low restraint scores. In line with previous work [25], it was expected that self-reported dieters would retrieve fewer specific memories than would non-dieters. Assuming the finding in dieters is due to concurrent restraint, it would be expected that the deficit in memory specificity in dieters would be larger in the group primed with healthy images.

## Methods

### Design

This study primarily used a 2 × 2 mixed factorial design, with priming condition (healthy vs unhealthy) as the between subjects’ factor and cue valence (positive vs negative) on the autobiographical memory test as the within subjects’ factor. A further 2 × 2 univariate design was used with dieting status and priming condition as the two between participant factors. The dependent variables were the time taken (in seconds) to retrieve specific memories and the proportion of specific memories retrieved.

### Participants

Sixty^1^ female undergraduate students (mean age = 24.6, SD = 4.8), recruited using posters and social media advertisements, took part in the study in exchange for course credit. Participants were assigned to one of two groups of 30. Ten individuals within each group reported that they were currently dieting. Characteristics of the two groups can be seen in Table 1. An a priori power calculation using G*Power revealed that a sample size of 56 (28 per priming condition) would be required to detect a medium effect size (based on a predicted partial eta squared of 0.05) with a power of 0.8 and an alpha level of 0.05, thus the study was adequately powered. The study was approved by Research Ethics Committee of De Montfort University.

**Table 1:** Participant characteristics (standard deviations are presented in parentheses)

	Healthy food primes (*n* = 30)	Unhealthy food primes (*n* = 30)	*t*-value	*P*-value	Cohen’s *d*
Age	24.33 (4.2)	24.9 (5.5)	0.45	> 0.05	0.11
BMI	26.41 (5.5)	24.57 (6.0)	1.19	> 0.05	0.32
Anxiety (HADS)	9.57 (4.0)	8.9 (4.5)	0.61	> 0.05	0.16
Depression (HADS)	4.6 (3.7)	4.1 (3.4)	0.58	> 0.05	0.15
Disinhibition (TFEQ)	6.53 (3.2)	7.3 (3.5)	0.92	> 0.05	0.23
Restraint (TFEQ)	8.67 (4.6)	9.03 (5.7)	0.27	> 0.05	0.08

*BMI* body mass index, *HADS* Hospital Anxiety and Depression Scale, *TFEQ* Three Factor Eating Questionnaire

### Materials and measures

#### Word cues

Six positive words (calm, lively, happy, glorious, lucky, excited) and six negative words (sad, upset, tired, bored, bad, awful) matched for emotionality, imageability, and frequency of usage were drawn from a previous autobiographical memory study [34].

#### Food images (primes)

Twenty-four food images (12 high calorie, e.g., pizza, and 12 low-calorie foods, e.g., salad) were drawn from a database of standardised food images (http://nutritionalneuroscience.eu/). The images had been rated for liking and perceived healthiness by adults and children [35]. The healthy (low-calorie) and unhealthy (high calorie) food depicted in the images used in the current study were equally liked, but high-calorie foods were rated as significantly less healthy than low-calorie foods.

#### Autobiographical memory test (AMT;[2])

Participants’ ability to retrieve specific memories of events from their past was assessed using a computerised variant of the AMT (presented using SuperLab version 5.1; Cedrus Corporation). Participants were presented with the word cues and invited to retrieve a specific memory in response to each cue word. A specific memory was defined as *“a memory of an event that occurred at a particular time and place and that lasted less than a day”.* Each word cue was primed with an image of a food item. Half of the participants were primed with images of healthy foods and half with images of unhealthy foods.

Each trial began with a focus point ( +) presented centrally (shown for 1 s), followed by a food prime (shown for 2 s^2^) and then a word cue (shown for 1 s) and participants asked to retrieve a specific memory in response to the cue word. Participants indicated they had retrieved a memory by pressing the space bar and were then asked to describe aloud the details of the memory, which were audio-recorded to allow for subsequent rating of specificity. If no memory was retrieved within 30 s (from the onset of the cue) then the next trial was initiated. Within each condition (healthy vs unhealthy) the order of the words and images was randomised for each participant. Prior to the main set of trials, participants completed two practice trials, where they received feedback relating to the specificity of their memory retrieval. Memories were coded as either specific (occurred at a specific time and place and lasted less than a day), categorical (summaries of repeated events), extended (events lasting longer than a day), or semantic associates (items, objects, places, or people associated with the cue). Decisions not to respond to a word, or failures to retrieve a memory within 30 s were coded as omissions. Memories were coded for specificity by the researcher who collected the data (either BV or JM) and subsequently by the first author (DW), who coded approximately 50% of the memories, to determine the reliability of the coding. There was a high degree of inter-rater reliability between the different coders (*K* = 0.85).

#### Hospital Anxiety and Depression Scale (HADS [36])

The HADS is a 14-item questionnaire designed to measure anxiety and depression, with 7 items relating to each construct. Each item consists of four statements pertaining to a particular symptom and the participant is asked to choose the statement that best represents how they have been feeling during the past week. Each item is scored from 0–3 based on increasing severity of negative mood (maximum score of 21 on each subscale). Cronbach’s α indicated good reliability in the present study (anxiety = 0.83; depression = 0.84).

#### Three Factor Eating Questionnaire (TFEQ;[37])

The 21-item cognitive restraint subscale from the TFEQ was used to determine the degree to which participants were restrained eaters (i.e. making conscious efforts to restrict their food intake). Twelve items, e.g., “I consciously hold back at meals in order not to gain weight” require a true/false response. Eight items, e.g., “How likely are you to consciously eat less than you want?” require a response using a 4-point Likert-type scale to indicate likelihood of this behaviour, and the final item requires participants to rate their degree of restraint from zero (eat what I want, whenever I want) to 5 (highly restrained: constantly limiting food intake, never ‘giving in’). For each item, a response indicating restrained eating is scored with 1 point, thus the range of possible scores on this scale is 0–21, with higher scores equating to greater restraint. The 16-item disinhibition subscale from the TFEQ was used to assess the tendency of participants to overeat according to habit, situation, or under challenging conditions. This measure was included as there is evidence that disinhibition is linked to impaired episodic recall [38]. The disinhibition subscale consists of 16 items; 13 items, e.g., “When I am blue, I often overeat” that require a true/false response and three items, e.g., “Do you eat sensibly in front of others and splurge alone?” that require a response on a 4-point Likert scale. Each response indicating a tendency to overeat is scored 1 point, thus the range of possible scores is 0–16 with higher scores equating to greater tendency towards disinhibited eating. In the present study both subscales showed good reliability, with Cronbach’s *α* of 0.74 (disinhibition) and 0.86 (cognitive restraint), respectively.

### Procedure

Having provided informed consent participants were randomly allocated to either the healthy or unhealthy priming conditions. They were then invited to complete the AMT followed by the demographic and questionnaire measures. Finally, height and weight were measured, in order to allow body mass index (BMI) to be calculated.

### Data analysis

Mean times (in seconds) to retrieve specific memories and the proportion of specific memories, adjusted for omissions, was calculated for each group and each type of word cue. For example, in response to positive cues if a participant retrieved four specific memories and made one omission then the proportion would be calculated using the following Eq. 4/(6–1) = , which would be 0.8 (80%). Retrieval times and proportion of specific memories were analysed using 2 prime (healthy vs unhealthy) × 2 cue valence (positive vs negative) mixed factorial ANCOVA, with HADS depression and TEFQ restraint scores as covariates. To examine the influence of dieting status on memory performance (overall memory specificity) a 2 prime (healthy vs unhealthy) x dieting status (dieting vs not-dieting) univariate ANCOVA was conducted with depression entered as a covariate. Relationships between individual difference variables and memory performance were assessed using Pearson correlations (α adjusted for multiple tests using Bonferroni correction).^3^

## Results

### Participant characteristics

Analysis of the participant characteristics (see Table 1) of the two priming groups (healthy vs unhealthy) revealed no significant group differences in age, BMI, depression, anxiety, restraint, or disinhibition (all tests *p* > 0.05). Dieters reported significantly higher restraint scores (mean = 13, SD = 3.6) than did non-dieters (M = 6.78, SD = 4.5); t(58) = 5.37, p < 0.001 (Cohen’s d = 1.47). However, they did not differ from non-dieters in age, BMI, depression, anxiety, or disinhibition (all tests p > 0.05).

### Autobiographical memory performance

Analysis of retrieval times for specific memories (presented in Table 2) revealed no main effects of valence, priming condition, restraint, or depression and no significant interactions, all tests *p* > 0.05.

**Table 2:** Mean retrieval times (in seconds) and proportion of specific memories as a function of priming condition and cue valence (standard deviations are presented in parentheses)

	Healthy primes (n = 30)		Unhealthy primes (n = 30)	
	Retrieval time (seconds)	Proportion of specific memories	Retrieval time (seconds)	Proportion of specific memories
Positive	11.81 (4.5)	0.62 (0.35)	11.28 (5.8)	0.81 (0.23)
Negative	12.62 (6.6)	0.65 (0.34)	11.32 (5.6)	0.73 (0.26)
Total	11.52 (5.17)	0.63 (0.35)	11.91 (6.1)	0.77 (0.25)

Analysis of the proportion of specific memories (see Table 2) revealed no main effects of valence; *F*(1, 54) = 0.23, *p* > 0.05, *η*^2^_*p*_ = 0.004, restraint; *F*(1, 54) = 0.15, *p* > 0.05, *η*^2^_*p*_ = 0.003, or depression; *F*(1, 54) = 0.44, *p* > 0.05, *η*^2^_*p*_ = 0.008. There was, however, a significant main effect of priming condition, such that participants primed with images of healthy foods retrieved significantly fewer specific memories (*M* = 0.63, SD = 0.35) than did participants primed with unhealthy images (*M* = 0.77, SD = 0.25); *F*(1, 54) = 4.45, *p* = 0.04, *η*^2^_*p*_ = 0.08. There was no significant condition x restraint interaction; *F*(1, 54) = 1.11, *p* > 0.05, *η*^2^_*p*_ = 0.02 and no condition x restraint x valence interaction; *F*(1, 54) = 1.49, *p* > 0.05, *η*^2^_*p*_ = 0.03. Importantly, there was no condition x depression nor condition x depression x valence interactions, both tests *F* < 1.

### Influence of dieting status on memory specificity

Analysis of the proportion of specific memories retrieved as a function of priming condition and dieting status revealed no main effect of dieting status, as dieters (*M* = 0.70, SE = 0.07) and non-dieters (*M* = 0.70, SE = 0.07) retrieved an equivalent proportion of specific memories; *F* < 1. There was also no main effect of depression; *F*(1, 55) = 0.11, *p* > 0.05, *η*^2^_*p*_ = 0.002 and no significant interactions, all tests *F* < 1. However, the main effect of condition in this analysis was trend significant; *F*(1, 55) = 2.89, *p* = 0.09, *η*^2^_*p*_ = 0.05.

### Relationships between individual difference factors and memory specificity

A series of Pearson tests (with Bonferroni adjusted *α* = 0.02) revealed that overall memory specificity was not related to anxiety, disinhibition, or BMI; all tests > 0.05.

## Discussion

The aim of the current study was to examine if using healthy and unhealthy food images to prime word cues on an autobiographical memory test would influence the proportion of specific memories retrieved on this task. As images of healthy foods were expected to make restraint salient [32, 33] and as restraint has been linked to reduced memory specificity [10, 26], it was predicted that, after controlling for depression, individuals primed with healthy images would retrieve significantly fewer specific memories than would participants primed with unhealthy images. This prediction was supported by the current findings. However, as priming of restraint should have been more evident in those with high restraint scores [32] it was expected that individuals with higher restraint scores in the healthy prime condition would exhibit poorer memory specificity than would those with low restraint scores. This prediction was not supported by the current data. The predictions that dieters would retrieve fewer specific memories than would non-dieters and that this difference would be larger in the group primed with healthy images were not supported by the current findings.

The finding that participants primed with healthy images produced fewer specific memories than did participants primed with unhealthy images could be due to the healthy images making restraint more salient, which in turn would have reduced the available resources available for the memory search [19–21]. However, the current finding that restraint was not related to specificity does not support this explanation. Similarly, the current finding that dieters did not retrieve fewer specific memories than did non-dieters is also inconsistent with this explanation, particularly as dieters reported significantly higher restraint than did non-dieters. Thus, it would appear unlikely that the observed difference in memory specificity was due to the salience of restraint. One alternative explanation concerns the possible influence of the different food items on the content of the memories retrieved. For example, it might be easier to recall specific memories of eating unhealthy foods (as these tend to be eaten at special occasions like birthdays) than healthy foods. However, examination of the content of the memories retrieved in the current study (*n*≈600) revealed that fewer than 10 memories referring to food were recalled, which does not support this explanation of group difference in specificity. Another plausible explanation concerns the affective response to the different food primes. Although the images were matched for liking [35], previous work has shown that images of unhealthy foods lead to greater increases in positive affect than do images of healthy foods [40] and are more rewarding than images of low-calorie foods [41]; thus, the current findings could be due to increases in positive affect in those primed with unhealthy images. Consistent with this proposal memory specificity has been linked to changes in positive affect [42].

The findings that restraint and dieting did not influence AMS are inconsistent with previous studies [25, 26]. It is possible that the variation in findings across the studies are due to differences in the type of memory cues utilised. In the current study we used positive and negative cue words (unrelated to eating), whereas Johannsen and Berntsen [25] used weight- and body-related words (e.g., food and clothes) and Ball et al. [26] utilised food-related (e.g., chocolate) and diet-related cues (e.g., exercise). It is, therefore, plausible that the cues used in the previous studies were more salient to dieters and restrained eaters and may therefore have been more likely to lead to impaired AMS [34]. This is important, as it shows that the relationship between restraint and memory specificity may be dependent on the salience of memory cues.

## Limitations

It is notable that we did not take a measure of state hunger, which potentially could have influenced the level of memory specificity. However, if this was the key factor then it would have been expected that images of unhealthy rather than healthy foods would have resulted in lower specificity, as, in hungry participants, images of high-calorie foods are more likely to attract attention [43, 44], and more likely to demand processing resources than healthy foods [45, 46]. Another limitation is that we did not compare primed with un-primed retrieval; this would have provided stronger evidence regarding if it was healthy images or unhealthy that led to changes in memory specificity. Another limitation is that we did not determine if the participants had a history of psychiatric diagnosis (e.g., depression or an eating disorder). However, in the advertisements for the study, it was requested that individuals with a history of a mental health condition should not volunteer for the study. Furthermore, the presence of individuals with clinically relevant depression, anxiety, or disordered eating would have been evident from the scores on the TFEQ and HADS (see Table 1), which were all within the normal range for the healthy population. Finally, it would have been useful to try to quantify changes in restraint and mood in response to the images. Future work should aim to confirm the current findings whilst addressing these limitations.

## Conclusions

As expected, priming autobiographical memory retrieval with images of healthy food led to lower memory specificity than did priming with unhealthy food primes. However, this cannot be explained in terms of increased salience of restraint, or current dieting behaviour, as neither of these factors was related to memory specificity. It would appear that the influence of restraint and dieting on memory specificity might be dependent upon cue salience. The most plausible explanation for the group difference in specificity is that unhealthy images might have led to an increase in positive affect relative to healthy images, which in turn led to greater memory specificity. The current findings suggest that priming might be a useful method of examining changes in memory retrieval that are related to eating behaviour.

## **What is already known on this subject?**

Dieting and dietary restraint are associated with reduced memory specificity, possibly due to reduced cognitive resources associated with restraint. Healthy food images have been shown to increase the salience of restraint. Therefore, priming word cues with healthy food images should lead to poorer memory specificity.

## **What this study adds?**

We aimed to provide novel evidence regarding the relationship between dietary restraint and autobiographical memory. We examined if priming restraint using images of healthy food reduced the number of specific memories recalled. In line with our expectations, participants who were primed with healthy images retrieved fewer specific memories. However, our results cannot be explained by restraint, as neither restraint nor dieting behaviour was linked to memory specificity. A plausible explanation for our findings is that unhealthy images may have led to an increase in positive affect, which in turn may have increased memory specificity. Our data suggest that the link between restraint and memory specificity is not robust. Further research is required to determine the conditions under which dietary restraint impairs memory specificity. For example, it might be a preoccupation with thoughts about dieting and weight that lead to reduced memory specificity rather than restraint alone.

## Data Availability

The dataset generated and analysed for this study can be requested from the corresponding author on reasonable request.

## Footnotes

1 An additional five participants took part, but were excluded from the data analysis due to concerns about the quality of their memory data—extremely fast RTs and very high numbers (e.g., 10/12) of omissions.

2 This timing was used to ensure supraliminal processing rather than subliminal.

3 Initial inspection of the data revealed that memory specificity, depression, age, and BMI were not normally distributed. Relationships were confirmed using non-parametric Spearman tests, but as the results were identical the results from the Pearson tests are reported. Given that ANOVA is robust to violations of normality (Schimder et al., [39]), we conducted the planned analyses. It should be noted, that are two samples showed homogeneity of variance.
